# Association of *TNF-alpha* Promoter Polymorphisms with Disease Susceptibility, mRNA Expression, and Lupus Nephritis in Mexican Patients with Systemic Lupus Erythematosus

**DOI:** 10.3390/jcm14113693

**Published:** 2025-05-25

**Authors:** Diana Celeste Salazar-Camarena, Claudia Azucena Palafox-Sánchez, Noemí Espinoza-García, Jorge Armando Guareña-Casillas, María Paulina Reyes-Mata, Jhonatan Velador-Mendoza, Miguel Marín-Rosales

**Affiliations:** 1 Grupo de Inmunología Molecular, Universidad de Guadalajara, Guadalajara 44340, Mexico; celeste.salazar@academicos.udg.mx (D.C.S.-C.); claudia.palafox@academicos.udg.mx (C.A.P.-S.); noemi.espinoza@academicos.udg.mx (N.E.-G.); paulina.reyes@academicos.udg.mx (M.P.R.-M.); 2 Hospital Civil de Guadalajara “Fray Antonio Alcalde”, Guadalajara 44350, Mexico; jorge.guarenac@academicos.udg.mx; 3 Doctorado en Ciencias Biomedicas, Universidad de Guadalajara, Guadalajara 44340, Mexico; jhonatan.velador9888@alumnos.udg.mx; 4 Secretria de Salud Jalisco, Hospital General de Occidente, Zapopan 45170, Mexico

**Keywords:** *TNFA* polymorphisms, TNF-α mRNA expression, sTNF-α, SLE

## Abstract

**Background/Objectives**: A case-control study was conducted to determine the association of the −238 G>A and −308 G>A *TNF-alpha* (*TNFA*) promoter polymorphisms with mRNA and protein expression in 180 Systemic Lupus Erythematosus (SLE) patients and 186 control subjects (CS) from western Mexico. **Methods**: Genotyping was performed using the PCR-RFLP method. *TNFA* mRNA expression was assessed by real-time quantitative PCR, and soluble TNF-α (sTNF-α) levels were quantified by ELISA. For comparison groups, Chi-square, Mann–Whitney U, or Kruskal–Wallis tests were used. Spearman’s rank correlation coefficient determined the correlation between variables. The Area Under the Curve was used to determine the diagnosing performance of sTNF-α. **Results**: No differences were found in the genotype distribution of −238 G>A and −308 G>A *TNFA* polymorphisms between SLE patients and CS. However, the −238A allele was associated with increased SLE susceptibility (OR 1.18 CI 95% 1.02–3.50, *p* = 0.037). Also, logistic regression analysis showed that LN risk was significantly higher in carriers of the −308A allele (OR 3.11 IC95% 1.15–6.43; *p* = 0.002). On the other hand, the *TNFA* mRNA expression was 3.3-fold higher in SLE compared to CS. SLE patients with −308 GG genotype showed higher *TNFA* mRNA expression compared to GA+AA genotype carriers (*p* < 0.01). Regarding sTNFa levels, SLE patients showed higher concentration than CS, mainly in lupus nephritis (LN), with a weak negative correlation with estimated Glomerular Filtration Rate and an acceptable accuracy for diagnosing SLE and LN, with areas under the curve of 0.61 and 0.65, respectively. **Conclusions**: The −238 A allele and −308 A allele of the *TNFA* gene are linked to a higher risk of susceptibility to SLE and LN in the western Mexican population. Additionally, SLE patients exhibited increased TNF-alpha gene expression and sTNF-α, particularly in LN, demonstrating acceptable diagnostic performance.

## 1. Introduction

Systemic Lupus Erythematosus (SLE; OMIM 152700) is the prototype autoimmune disease that involves immune complex deposition in nearly all tissues and organs, leading to a wide range of clinical symptoms [1]. The pathogenesis of SLE is multifactorial, including genetic and environmental factors [2]. Genetic predisposition plays a significant role in susceptibility [3], whereas environmental exposure can trigger the activation of the innate and adaptive immune response [4], leading to an irreversible loss of immunologic self-tolerance that induces or accelerates the development of SLE in susceptible individuals. Important advances in this regard have been made thanks to genome-wide association studies (GWAS) using hundreds of thousands of single nucleotide polymorphism (SNP) markers [5]. Among genetic factors believed to affect susceptibility to SLE, the major histocompatibility complex (MHC) alleles show the most significant association. Class III genes of the MHC encode proteins not involved in antigen presentation [6,7]. Tumor necrosis factor alpha (TNF-α) is a cytokine involved in systemic inflammation; it stimulates the acute phase response and increases MHC class I and II expression and antigen-driven lymphocyte proliferation [8]. Activated macrophages are the main source of TNF-α, although it can be produced as a trimer on the surface of many other cell types, such as lymphocytes and dendritic cells [9]. After processing by TNF-α-converting enzyme (TACE), the soluble form is cleaved from transmembrane TNF-α and mediates its biological activities through binding to type 1 and 2 TNF receptors (TNFR1 and TNFR2) in remote tissues [10]. TNF-α exerts functions that strongly contribute to inflammatory and immune responses by inducing various inflammatory cytokines and chemokines [11]. It has been suggested that serum levels of TNF-α are higher in SLE patients and correlate with disease activity [12,13]. Moreover, TNF-α was highly overexpressed in both sera and renal tissue of MRL/lpr lupus mice, and the levels of TNF correlated with the degree of inflammatory organ disease [14].

The *TNFA* gene is located on chromosome 6p21.3, within the class III region of MHC. Several Single Nucleotide Polymorphisms (SNPs) have been identified inside the *TNFA* promoter. The substitution of adenine for guanine (G>A) at positions −238 (rs361525) and −308 (rs1800629) directly affects gene regulation, and these gene variants have been associated with the altered transcriptional activity of *TNFA* in various disorders [15]. The −238A allele was reported to down-regulate *TNFA* expression [16], whereas the −308A allele correlates with higher transcriptional activity [17,18]; however, some other studies reported inconsistent results. Although these gene variants have been widely studied in many populations, it has been demonstrated that differences in risk gene variants exist across different continental populations. Regarding Mexican mestizos, Ramírez-Bello et al. reported the association between SLE and lupus nephritis with other *TNFA* gene polymorphic variants (−1031 T>C and −376G>A) [19]. However, the Mexican population has rich diversity of source ancestry, with Southern European, Amerindian, and West African contributions to the inherited genome [20]. Also, the functional effect of −238 G>A and −308 G>A *TNFA* polymorphisms justifies studying their impact on *TNFA* gene expression and clinical phenotypes in SLE patients from western Mexico. Therefore, the present study aimed to investigate the relationship between the −238 G>A and −308 G>A *TNFA* promoter gene SNPs to SLE susceptibility in western Mexican patients and whether these genetic variants are associated with the mRNA expression and clinical and immunological features of the disease, focusing on LN.

## 2. Materials and Methods

### 2.1. Patients and Controls

The present case-control study included 366 Mexican mestizos defined by the National Institute of Anthropology and History [20]. One hundred and eighty patients with SLE fulfilling the 1997 revised American College of Rheumatology criteria and/or 2012 Systemic Lupus International Collaborating Clinics criteria were recruited from the Department of Rheumatology and Immunology from Hospital General de Occidente, Mexico. Additionally, 186 unrelated control subjects (CS) were collected from the general population; these subjects were blood donors with no history of autoimmune disorders who were gender and age-matched. The Mexican versions of the Systemic Lupus Erythematosus Disease Activity Index (Mex-SLEDAI) [21] and SLICC Damage Index (SLICC-DI) [22] were evaluated in all SLE patients at the enrollment.

### 2.2. Ethics Statement

The local research ethics committees approved the study (approval number CI 292/23). All patients provided written informed consent before participating in the study. All clinical investigations were conducted according to the principles expressed in the Declaration of Helsinki.

### 2.3. Genotyping of −238 and −308 TNFA Polymorphisms

According to the salting-out method, genomic DNA (gDNA) was extracted from 5 mL of peripheral blood cells. *TNFA* polymorphisms were identified using Polymerase Chain Reaction (PCR) followed by Restriction Fragment Length Polymorphisms (RFLP). Primer sequences for *TNFA* −238 G/A (rs361525) were forward: 5′-AGA AGA CCC CCC TCG GAA CC-3′ and reverse: 5′-ATC TGG AGG AAG CGG TAG TG-3′; for *TNFA* −308, G/A (rs1800629) were forward: 5′-AGG CAA TAG GTT TTG AGG GCC AT-3′ and reverse: 5′-TCC TCC CTG CTC CGA TTC CG-3′. For both forward primers, a design containing a single base-pair mismatch adjacent to the polymorphic site was made to introduce a restriction site into the wild-type nucleotide sequence during the amplification reaction. The PCR was carried out at a final volume of 12.5 μL: 1.25 μL of 10X supplied buffer enzyme, 2.5 mM of MgCl_22_, 2.5 mM of each dNTP, 0.025 U/μL of Taq DNA polymerase (Invitrogen Life Technologies, Carlsbad, CA, USA), and 30 ng/μL of gDNA. The same amplification protocol was performed: initial denaturalization at 94 °C, followed by 33 cycles of 94 °C for 30 s, 60 °C for 30 s, and 72 °C for 30 s, with a final extension of 72 °C for 2 min (Techne TC-5000). The PCR yields a 152 bp for *TNFA* −238 and 107 bp for *TNFA* −308. Amplification products were digested (New England BioLabs, Beverly, MA, USA) with 3 U of *MspI* (for *TNFA* −238) or *NcoI* (for *TNFA* −308) at 37 °C for 1 h. Restriction fragments were separated in electrophoresis 6% gel polyacrylamide and stained with 2% silver nitrate. The wild-type *TNFA* −238 G allele generates site recognition for the endonuclease *MspI*, obtaining 133 bp and 19 bp fragments. Otherwise, the A allele prevents the recognition sequence for the endonuclease, and the 152 bp remains intact. For *TNFA* −308, the wild-type G allele is recognized by the endonuclease *NcoI*, which generates 87 bp, 20 bp, and 107 bp fragments, representing the *TNFA* −308 A allele.

### 2.4. RNA Extraction and Reverse Transcription

Total cellular RNA was extracted from peripheral blood mononuclear cells (PMBCs) using Trizol reagent (Invitrogen Life Technologies, Carlsbad, CA, USA) according to the manufacturer’s instructions. Repeated phenol–chloroform extraction of RNA samples was subjected to isolation of the RNA according to the Chomiczyki and Sacchi method [23]; the ratio was used to provide an estimate of the purity of the nucleic acid, and in all samples ranged between 1.8 and 2.0. The RNA integrity was corroborated on 1X TBE agarose gel. The samples with low-quality and degraded RNA were excluded from the study. Complementary DNA (cDNA) was synthesized from 2 μg of total RNA using oligo-dT and GoScript™ Reverse Transcription System (Promega Corp., Madison, WI, USA) following the manufacturer’s protocol. The cDNA samples were stored at −80 °C until the real-time PCR assays. The *TNFA* mRNA expression was determined in thirty-five SLE patients and fifteen CS carrying different genotypes for rs361525 and rs1800629 promoter polymorphism.

### 2.5. Quantitative PCR (qPCR)

Quantitative real-time polymerase chain reaction (qPCR) was carried out to quantify the gene expression of interest. The RT-qPCR protocol followed the guidelines of the Minimum Information for Publication of Quantitative Real-Time PCR Experiments (MIQE) [24]. The reaction was run on a Nano Light Cycler 2.0 (Roche Applied Science, Penzberg, Germany). Glyceraldehyde-3-phosphate dehydrogenase (GAPDH) was used as a reference gene to determine relative quantification after being shown to be stably expressed in the sample [25]. The primers and hydrolysis probes were designed on the Roche Universal Probe Library software version 2.1 (TNFA: cat. no. 04688546001, GAPDH: probe cat. no. 05190541001), and the primers were validated by gel electrophoresis. All samples were run as triplicates. After validating the PCR efficiencies for both genes, the obtained data were analyzed. A comparative threshold cycle (Ct) method with a cut-off of 40 cycles was used to determine the TNFA mRNA copy number relative to GAPDH based on the 2^−ΔΔCt^ method [26] and 2^−ΔCt^ method [27].

### 2.6. TNF-α Serum Levels Determination

Serum TNF-α levels were determined in a Multiskan GO spectrophotometer (Thermo Fisher Scientific Oy, Ratastie, PO, Finland) using a commercial ELISA (cat. no. 430204, BioLegend, San Diego, CA, USA) with a sensitivity of 2.0 pg/mL and 7.8–500 pg/mL standard range.

### 2.7. Data Analysis

Categorical variables were presented as absolute values and percentages, and continuous variables were expressed as median and interquartile range (IQR). Allele and genotype frequencies were obtained by direct counting. Concerning the evaluation of *TNFA* gene polymorphisms, the significance of differences from the Hardy–Weinberg equilibrium (HWE) was tested using the χ^2^ test or Fisher’s exact test. Odds ratios (OR) and 95% confidence intervals (95% CI) were calculated to evaluate the risk of SLE associated with the *TNFA* promoter polymorphisms. Kruskal–Wallis, Mann–Whitney U, and Spearman’s rank correlation coefficient were used to compare serum TNF concentrations with SLE clinical variables. Statistical analyses were performed with SPSS v21 (IBM Corporation; Armonk, NY, USA) and GraphPad Prism 10.3.0 (GraphPad Software, Incorporation; La Jolla, CA, USA) software. Differences were considered significant with a *p*-value < 0.05.

## 3. Results

### 3.1. Demographical Data and Clinical Features

A total of 366 participants (15 males, 351 females) were selected, including 180 SLE patients and 186 CS. The median age and gender distribution were similar, and no statistical difference was observed between the groups (*p* > 0.05). In the SLE group, 171 patients were female (95%), and the median age was 34 (IQR 26–45.5). The disease evolution was five years (IQR 2–11) in SLE patients. Regarding Count Blood Cells (CBC), most results were within the reference values. However, when comparing specific parameters, the SLE patients showed a slightly lower count, with a statistically significant difference (Leukocytes [6.7 × 10^3^/μL (IQR 5.8–7.9) vs. 5.3 × 10^3^/μL (IQR 4.2–6.8) *p* < 0.001], Hemoglobin [14.6 g/dL (IQR 13.9–15.2) vs. 12.8 g/dL (IQR 11.7–14), *p* < 0.001], and Platelets [243 × 10^3^/μL (IQR 243–342) vs. 254 × 10^3^/μL (IQR 209.5–307.1) *p* < 0.001], respectively).

The median disease activity index (Mex-SLEDAI) was 2 (1–4), and the organ damage index (SLICC) was 0 (IQR 0–1). The most frequent clinical domains were haematological (60.2%), mucocutaneous (30.5%), and articular (18%). Concerning kidney involvement, the estimated Glomerular Filtration Rate (eGFR) was evaluated in 122 cases, with an eGFR of 98.5 mil/min (IQR 68.5–117.5). Clinically active lupus nephritis (LN) was present in 15.6% of cases (27/173). The LN group had a lower eGFR of 75 mil/min (IQR 27–112.5) than the no-LN group [107 mil/min (IQR 86–122), *p* = 0.0449].

### 3.2. Genotypic and Allelic Distribution of −238 (rs361525) and −308 (rs1800629) G>A TNFA Polymorphisms

The distributions of allele and genotype frequencies of the polymorphisms in the studied individuals are depicted in Table 1. The distribution of genotypes for the −238 G>A and −308 G>A polymorphisms in healthy controls was in accordance with the Hardy–Weinberg equilibrium (*p* = 0.531 and *p* = 0.391). The TNFA −238 G>A and −308 G>A genotypes did not show statistical differences between groups. The frequency of the −238A allele (8.3%) was significantly increased in SLE patients compared with controls (4.3%) (OR = 1.89, 95%CI: 1.02–3.15, *p* < 0.05). Differences were not found in dominant or recessive inheritance models (Table 1). For −308 G>A polymorphism, the allelic frequencies in the SLE and CS groups were as follows: 94 vs. 90.3% for the −308G allele and 9.7% vs. 6% for the −308A allele, and no significant differences were observed.

### 3.3. Genotype Distribution in SLE and Association with Lupus Nephritis

In order to determine the association of TNFA promoter polymorphism and lupus nephritis (LN), we classified genotype and allele frequencies according to the outcome variable LN, defined as fulfilling the ACR classification criteria for the renal manifestation of SLE (persistent proteinuria > 0.5 g per day or greater than 3+ by dipstick, and/or cellular casts) or evidence of LN on kidney biopsy [28]. The univariate logistic regression analysis showed that the variant allele harbors the risk of −308 AA and −308 GA genotypes, yielding 9.38-fold (95% CI = 1.020–107.4, *p* = 0.030) and 2.86-fold (95% CI = 1.212–6.785, *p* = 0.013) increased risk of LN. Therefore, the −308A allele was more frequent in the LN group than in the Non-LN group, Table 2. The relation between −238 G>A and LN was not observed.

### 3.4. TNFA mRNA Expression

**Figure 1:**
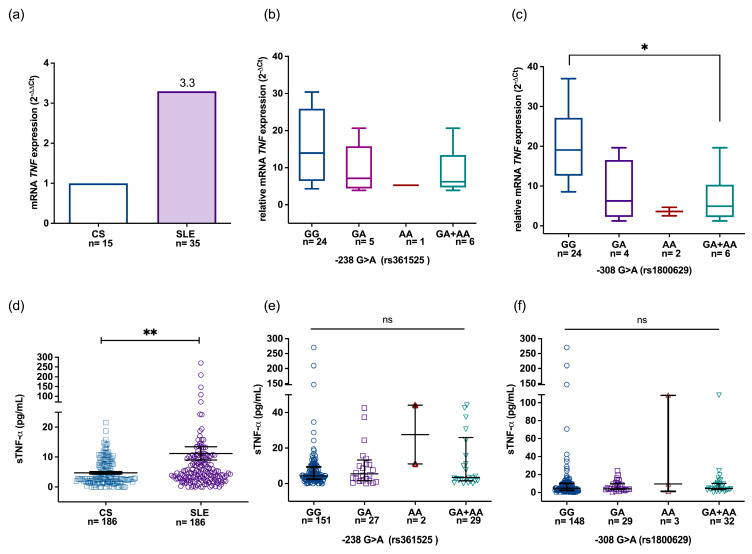
mRNA expression and soluble TNF-α levels in CS and SLE patients. Relative expression of TNFA mRNA in PMBCs of CS and SLE determined by the 2^−ΔΔCt^ method (**a**). A stratification analysis was performed according to the promoter TNFA −238 GA and −308 GA genotypes determined by the 2^−ΔCt^ method. TNFA mRNA in PMBCs of SLE in representative SLE carriers grouped by dominant genetic model (**b**,**c**). Soluble TNF-α levels in CS and SLE patients (**d**) and the corresponding soluble TNF-α levels according to the −238 GA (**e**) and TNF −308 GA (**f**) genotypes, respectively. Lines depict the median and interquartile range (IQR). ** *p* < 0.01, statistical comparisons between groups were made using the Mann–Whitney U test. * *p* < 0.01, Kruskall–Wallis test with Dunn’s correction.

To know if the TNFA polymorphisms are associated with gene expression, the TNFA mRNA levels were determined in SLE patients and CS, carriers of different genotypes. In peripheral blood, TNFA gene expression was 2.3-fold higher in patients with SLE compared to CS (Figure 1a) using the 2^−ΔΔCt^ method. When the mRNA expression in SLE patients was analyzed based on −238 genotypes or genetic inheritance models using the 2^−ΔCt^ method, no statistical differences were observed (Figure 1b). However, regarding −308 G>A polymorphism, in the dominant model, carriers of −308A allele (GA+AA) showed a lower TNFA mRNA expression [4.95 (IQR 2.18–10.35)] compared to the homozygous GG genotype [19.05 (IQR 12.58–27.13); *p* = 0.015] (Figure 1c).

### 3.5. Soluble TNF-α Levels

As shown in Figure 1d, the median of TNF-α serum levels in SLE patients was 3.43 pg/mL (IQR 2.66–10.04), which was significantly elevated compared to CS [2.82 pg/mL (IQR 1.85–7.6) *p* < 0.001]. However, no differences were found when soluble levels were compared in SLE patients according to −238 and −308 TNFA genotypes and inheritance models (Figure 1e and Figure 1f, respectively).

### 3.6. Association Between the Frequencies of the Dominant Model of rs1800629 (−308 G>A) and Clinical Domains in SLE

**Figure 2:**
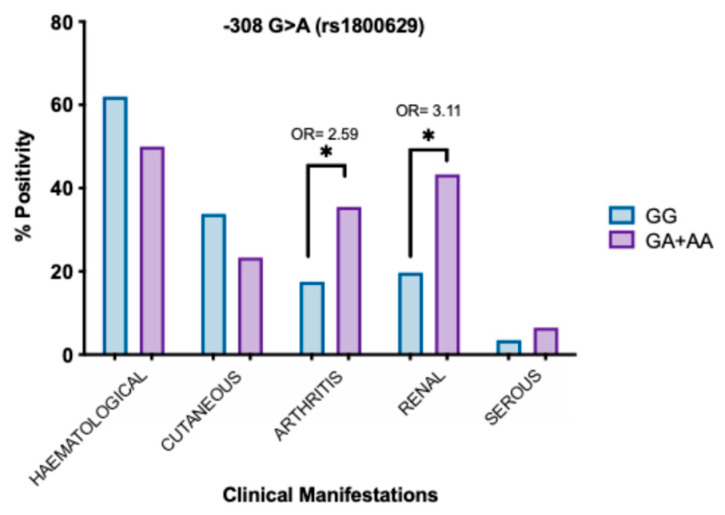
Association of clinical manifestations and dominant model of rs1800629. Comparison of GG and GG+AA genotypes of rs1800629 according to clinical manifestations. *p*-value was obtained through the Chi-square test. OR: odds ratio. * *p* < 0.05.

Subsequently, we grouped the genotype frequencies according to the dominant model of rs1800629 polymorphism and compared the clinical manifestations of SLE patients. SLE patients carriers of GA+AA genotypes showed a higher risk of developing kidney involvement [OR 3.11 (95% CI 1.35–7.16), *p* = 0.005] and arthritis [OR 2.59 (95% IC), *p* = 0.024]. No statistically significant differences were detected in other domains (Figure 2).

### 3.7. Clinical Association and Diagnosis Performance of Soluble TNF-α Levels in SLE Patients

**Figure 3:**
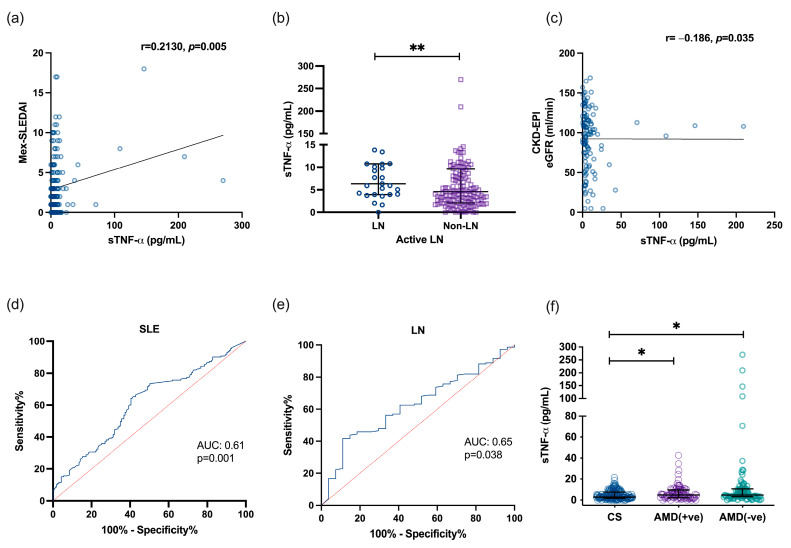
Association between and clinical accuracy of soluble TNF-α levels in SLE, LN, and use of antimalarial drugs. Correlation of disease activity index and sTNF-α (**a**). Comparison of sTNF-α between SLE patients with LN and Non-LN (**b**), correlation of sTNF-α with eGFR (CKD-EPI) (**c**), sTNF-α diagnosis performance SLE (**d**), sTNF-α diagnosis performance of LN (**e**), and comparison of sTNF-α between CS, and SLE patients with and without AMD (**f**). ** *p*< 0.01, statistical comparisons between groups were made using the Mann–Whitney U test. * *p* < 0.01, Kruskall–Wallis test with Dunn’s correction. Abr: SLE: Systemic Lupus Erythematosus, LN: lupus nephritis, and eGFR: estimated Glomerular Filtration Rate.

In SLE patients, high levels of soluble TNF-α were observed, which correlated positively with the disease activity index (r = 0.213, *p* = 0.005; Figure 3a). Additionally, higher concentrations of this cytokine were associated in patients with LN (Figure 3b). There was also a negative correlation between soluble TNF-α levels and eGFR (r = −0.1859, *p* = 0.0349; Figure 3c). Based on these findings, we investigated the clinical utility of soluble TNF-α in diagnosing SLE and LN. The cytokine showed acceptable accuracy for diagnosing both conditions, with an Area Under the Curve (AUC) of 0.61 (*p* = 0.001; Figure 3d) and an AUC of 0.65 (*p* = 0.038, Figure 3e), respectively. On the other hand, the treatment with antimalarial drugs (AMD) did not show an association with the concentration of sTNF-α (Figure 3e).

## 4. Discussion

Although the etiology of SLE remains elusive, the contribution of the genetic component to disease susceptibility is recognized [5]. Genes of the MHC are considered major contributors to an autoimmune response [7]. TNF-α is a cytokine that contributes to the pathogenesis of SLE and could amplify the immune response by activating inflammatory and endothelial cells. It also enhances the expression of adhesion molecules, IL-1, IL-6, and IL-18 [13], promotes T-cell activation, and could be associated with an imbalance between regulatory T cells and Th17 cells, and autoantibody production by B cells [13,29].

The promoter region polymorphisms −238 G>A and −308 G>A have been linked to a wide variety of MHC-associated autoimmune diseases [15,30,31], including increased risk of SLE [32,33,34,35]. However, some authors have not found associations [36,37,38]. The study of these SNPs of the *TNFA* gene in the western Mexican population has been considered relevant because the ancestry distribution in countries, even in Mexican cities, was different. Also, informative ancestry markers can help explore the relationship between specific genes and ancestry in the development of Systemic Lupus Erythematosus among various ethnic groups [39].

This study evaluated the −238 G>A and −308 G>A *TNFA* polymorphisms in SLE patients and CS from western Mexico. The genotype distribution for the SNPs analyzed was similar in both study groups. The frequency of the GA genotype of both polymorphisms shows a slight increase in western CS concerning other Mexico cities (9.1% vs. 6.9% and 11.8% vs. 8.7%, respectively) [19].

The allele frequencies in −238 G>A and −308 G>A *TNFA* polymorphisms were similar to those previously reported in RA and SLE Mexican patients [19] and those reported in the India, South America, and North American SLE populations [30,38,40].

In this study, the −238A allele was significantly associated with an increased risk of SLE development. In a previous study in the Mexican population, linkage disequilibrium between *TNFA* −238 and DRB1*1401 and DRB1*0301 alleles was observed in SLE patients. This finding suggests that the association between −238 *TNFA* and SLE could result from this linkage disequilibrium between *TNFA* and DRB alleles in Mexican SLE patients [41].

LN is one of the most critical life-threatening manifestations of lupus because 10–20% of patients could progress to end-stage renal disease within five years of diagnosis [42]. This study found an association between carriers of the 308A allele and lupus nephritis; however, this could be controversial in Mexican lupus nephritis patients. Recently, Ramirez-Bello et al. found no association with LN concerning the −308 G>A *TNFA* gene variant; however, they found an association between the −376 G>A polymorphism and LN [19]. This finding could be explained through the ancestry diversity in the Mexican population [43]. On the other hand, Piotrowski et al. and Yang et al. reported the association of *TNFA* −308 A allele with kidney involvement in the Polish, European, and Chinese SLE populations [44,45].

The mRNA expression levels showed a 2.3-fold increase in PMBCs of SLE patients, similar to the findings in previous studies that reported elevated *TNFA* transcription in SLE patients [46,47]. Furthermore, the relationship between the −238 and −308 *TNFA* genotypes and gene expression was analyzed. While the polymorphism −238 G>A of the *TNFA* gene showed no association with the mRNA expression, for the carriers of the −308 GG genotype increased expression of the *TNFA* gene was observed. This finding is in agreement with some studies previously published [48,49]. However, the discrepancy remains regarding the functional aspect of this SNP.

Wilson et al. conclude that the G to A nucleotide change at −308 positions modifies the consensus sequence for the transcription factor AP-2 binding site, forming an altered composite transcriptional element [18,50] with higher mRNA production from −308A allele in reporter gene assays [16]. However, it is important to note that there are many biological steps, in addition to the influence of genetic polymorphisms in the control of the production and release of TNF-α.

Regarding soluble TNF-α, SLE patients showed higher concentrations than CS, mainly those patients with kidney involvement. The association of this cytokine with several autoimmune diseases has been previously described, including SLE with LN [12,29,51]; however, the TNF-α participation in this condition has been debated. Even so, the concentration of this biomarker in our study was similar to that reported by Idborg et al. in Swedish SLE patients; however, the discriminatory capacity of this biomarker was lower [52]. The TNF-α has immunoregulatory and proinflammatory functions on a range of cells. Also, TNF-α is a growth factor for B lymphocytes and promotes the production of IL-1 and IL-6 [53]. Also, the B cells can produce significant amounts of TNF-α in an autocrine loop [54].

The role of TNF-α in lupus nephritis is supported by the evidence of the increased expression of TNF-α in the glomeruli; high urinary levels; and the activation of the complement cascade with and without specific TNF gene polymorphisms in the affected patients [55]. In addition, TNF-α exerts its function through the TNF-α receptor I (sTNF-RI), and this axis plays an essential role in the physiopathology of SLE and LN. Regarding sTNF-RI, Liu et al. reported the association of this receptor with lupus disease activity and LN, proposing it as a biomarker of LN [56]. However, multiple cytokines are involved in the physiopathology of LN, so several mechanisms could contribute to damage progression [42]. Additionally, anti-TNF therapy has been successful in other kinds of autoimmune diseases like Rheumatoid Arthritis, Psoriatic Arthritis, and Spondyloarthropathies. However, the use of this biological therapy in SLE has been controversial. Paradoxically, the anti-TNF blockage therapy could generate autoantibodies and SLE-like clinical manifestations [10].

## 5. Conclusions

The present study suggests that the allele A of −238 G>A polymorphism and allele A of −308 polymorphism of the *TNFA* gene are associated with SLE susceptibility and LN in the western Mexican population, respectively. Furthermore, *TNFA* gene expression is associated with LN, and sTNF-α is associated and correlated with disease activity, with an acceptable accuracy diagnosis of SLE and LN. However, more studies are necessary to elucidate this finding.

## Figures and Tables

**Table 1:** Genotypic and allelic frequencies of the TNFA polymorphisms in SLE patients and control subjects.

		SLE (180) n (%)	CS (186) n (%)	*p*-Value	OR (95% CI)	*p*-Values for HWE Test in Controls
−238 G>A rs361525	GG	152 (84.5)	169 (90.9)	1	-	0.513
	GA	26 (14.4)	17 (9.1)	0.106	1.70 (0.88–3.25)	
	AA	2 (1.1)	0 (0)	0.137	5.56 (0.27–116.75)	
	G	330 (91.7)	355 (95.4)	1	-	
	A	30 (8.3)	17 (4.6)	**0.037**	**1.89 (1.02–3.50)**	
	^†^ GG	152 (84.4)	168 (90.9)	1	-	
	GA+AA	28 (15.6)	17 (9.1)	0.061	1.83 (0.96–3.47)	
	^‡^ GG+GA	178 (98.9)	186 (100)	1	-	
	AA	2 (1.1)	0 (0)	0.149	0.19 (0.01–4.01)	
−308 G>A rs1800629	GG	148 (82.2)	164 (88.2)	1	-	0.391
	GA	29 (16.1)	22 (11.8)	0.211	1.46 (0.80–2.65)	
	GA	3 (1.7)	0 (0)	0.069	7.75 (0.39–151.36)	
	G	325 (90.3)	350 (94)	1	-	
	A	35 (9.7)	22 (6)	0.054	1.71 (0.98–2.98)	
	^†^ GG	148 (82.2)	164 (88.2)	1	-	
	GA+AA	32 (17.8)	22 (11.8)	0.108	1.61 (0.89–2.89)	
	^‡^ GG+GA	177 (98.3)	186 (100)	1	-	
	AA	3 (1.7)	0	0.077	0.14 (0.01–2.65)	

SLE, systemic lupus erythematosus; CS, control subjects; OR, odds ratio; 95% CI, 95% confidence intervals; and HWE, Hardy–Weinberg equilibrium. ^†^ Dominant model of inheritance; ^‡^ recessive model of inheritance. The values are presented as frequency in percentage and number of genotypes or alleles. The frequency comparison between groups was analyzed using Chi-Square or Fisher’s exact test. Results that are highlighted in bold indicate a statistically significant difference. Statistical significance value *p* ≤ 0.05.

**Table 2:** Comparison of Genotypic and allelic frequencies of the TNF polymorphisms in LN nephritis and non-LN patients.

		LN (39) n (%)	Non-LN (141) n (%)	OR (95% CI)	*p*-Value	*p*-Ajusted
−238 G>A rs361525	GG	33 (84.6)	119 (84.4)	1	-	
	GA	5 (12.8)	21 (14.99)	0.85 (0.301–2.451)	0.775	
	AA	1 (2.6)	1 (0.7)	3.60 (0.220–59.21)	0.337	
	G	71 (91.0)	259 (91.8)	1	-	
	A	7 (9.0)	23 (8.3)	1.11 (0.458–2.692)	0.816	0.821
−308 G>A rs1800629	GG	26 (66.7)	122 (86.5)	1	-	
	GA	11 (28.2)	12 (12.8)	**2.86 (1.212–6.785)**	**0.013**	
	GA	2 (5.1)	1 (0.7)	**9.38 (1.020–107.4)**	**0.030**	
	G	63 (80.8)	262 (92.9)	1	-	
	A	15 (19.2)	20 (7.1)	**3.11 (1.513–6.432)**	**0.001**	**0.002**

Non-LN, non-lupus nephritis; OR, odds ratio; and 95% CI, 95% confidence intervals. The values are presented as frequency in percentage and number of genotypes or alleles. The frequency comparison between groups was analyzed using Chi-Square or Fisher’s exact test. Results that are highlighted in bold indicate a statistically significant difference. Statistical significance value *p* ≤ 0.05.

## Data Availability

The original contributions presented in this study are included in the article. Further inquiries can be directed to the corresponding author.
